# Trialling the Hailie® smart inhaler with children and young people for asthma management in the United Kingdom: A nested qualitative evaluation

**DOI:** 10.1177/20552076251378435

**Published:** 2025-10-23

**Authors:** Irtiza Qureshi, Mayuri Gogoi, Manish Pareek, Hilary Pinnock, David Lo, Tony Bowden, Sander ten Veldhuijs, Jacqui Melville, Erol A Gaillard

**Affiliations:** 1 Department of Respiratory Sciences, 6123 University of Leicester, Leicester, UK; 2 Development Centre for Population Health, 4488 University of Leicester, Leicester, UK; 3 Centre for Inequalities, Institute for Lifecourse Development, 4918 University of Greenwich, London, UK; 4 Nottingham Centre for Public Health and Epidemiology, University of Nottingham, Nottingham, UK; 5 Department of Infection and HIV Medicine, University Hospitals of Leicester NHS Trust, Leicester, UK; 6 National Institute of Health Research (NIHR) Leicester Biomedical Research Centre, Leicester, UK; 7 National Institute of Health Research (NIHR) Applied Health Collaboration (ARC) East Midlands, UK; 8 Asthma UK Centre for Applied Research, Usher Institute, University of Edinburgh, Edinburgh, UK; 9 Helicon Health, London, UK; 10 Adherium, Melbourne, Australia

**Keywords:** Digital monitoring, primary care, paediatric, acceptability, adherence, inhaler

## Abstract

**Background:**

Monitoring of inhaler use in high-risk children has the potential to reduce asthma attacks and asthma-related deaths. We, therefore, undertook the first UK primary care study to identify high-risk children and young people by searching primary care health records and provided the Hailie® smart inhaler to monitor their asthma medication usage. In this article, we present data from the nested qualitative study, conducted with key stakeholders.

**Methods:**

This qualitative interview-based study explored a range of topics relating to the experiences of paediatric asthma care and management, including the use of the Hailie® smart inhaler, from the perspectives of the children, their parents/carers and healthcare professionals. Interview transcripts were generated and thematically analysed.

**Results:**

Six parent–child dyads and one parent were interviewed, either online or face-to-face. Additionally, three healthcare professionals (1 Nurse, 1 Pharmacist and 1 Practice Manager) involved in paediatric asthma care and/or management were also interviewed. Two specific themes were identified: Firstly, app-based monitoring was generally viewed positively and was reassuring to parents. Children also appreciated learning about using their inhalers. Secondly, challenges with synching were identified and users had some practical suggestions for improvement. Healthcare professionals also observed that monitoring should not replace clinical support for self-management.

**Conclusion:**

Our findings support the acceptability and usefulness of the Hailie® smart inhaler amongst children with high-risk asthma, although some technical difficulties need to be addressed. Further research is needed to assess effectiveness in clinical care management.

## Introduction

Around one million children and young people (CYP) in the United Kingdom (UK) live with chronic asthma, the most common non-communicable long-term medical condition in UK CYP.^1^ On average, one child dies from asthma every 4 weeks in the UK.^2^ The death rate for children living in the most deprived neighbourhoods of England is four times higher compared to the least deprived and it was identified that the majority of children who died had experienced at least one severe asthma attack in the year prior to their death.^2^ These children are also less adherent to their preventer inhaler and collect excessive prescriptions for salbutamol rescue inhaler.^2^

Deaths associated with asthma are considered to be largely preventable with improved management and early intervention.^3^ Smart inhalers can improve adherence to preventer medication and clinical outcomes, and it has been shown that electronic monitoring of inhaler use in high-risk populations has the potential to reduce asthma attacks and asthma-related deaths.^3,4,5,6^ Studies involving CYP with asthma managed in primary care are lacking and this is important because the majority of CYP dying from asthma have never been seen in specialist care.^7^

**Figure 1.**
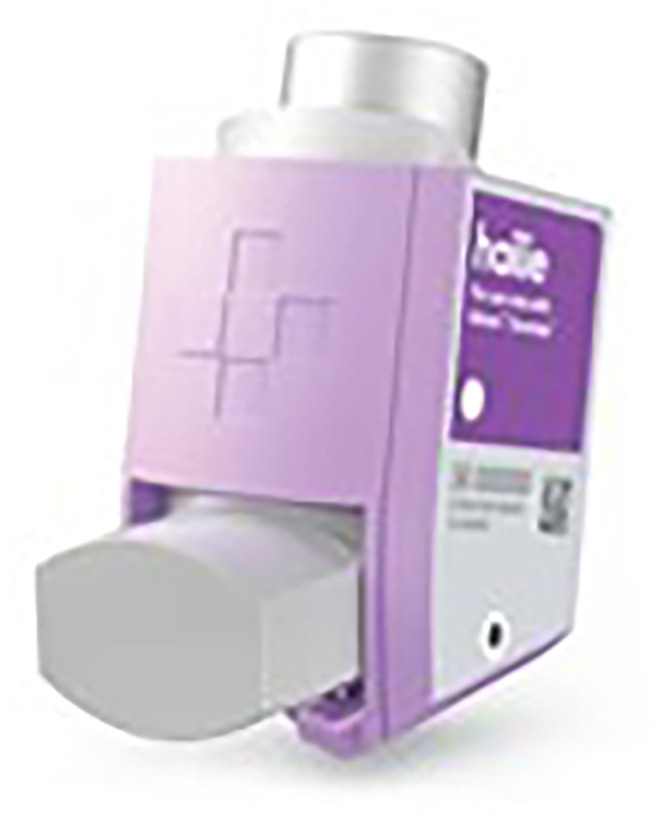
The Hallie® smart inhaler.

In recent years, there have been several technological developments which aim to support patients with asthma.^8^ The Hailie® smart inhaler is an example of one such development (see Figure 1).

The Hailie® smart inhaler attaches onto the CYP's usual inhaler and provides audible reminders to facilitate adherence. It monitors inhaler use and technique and provides feedback to the child and/or their family via a smartphone app. Simultaneously, the child's medical team can access the data online, enabling them to monitor and support families in improving adherence and inhaler technique, with the aim of enhancing symptom control and reducing the risk of exacerbations.

Our study is the first in UK primary care to deploy the Hailie® smart inhaler device to CYP living in Leicestershire with poorly controlled asthma and at high risk of an acute attack. The overarching objective of the study was to test the acceptability and feasibility of remote digital smart inhaler monitoring in high-risk children treated for asthma in primary care. In this paper, we present findings from the nested qualitative study, where we interviewed parents (For parents please read parent and or carer), young people and healthcare providers (HCPs), who were involved in the main study, about their perceptions and experiences of digital smart inhaler monitoring.

## Methods

We conducted a prospective observational cohort study in children aged 5–16 years with high-risk asthma managed in Leicestershire/UK primary care between November 2023 and December 2024. For the main study, high risk patients in participating practices were identified from their primary care records, and defined as those with poor asthma control (multiple reliever prescriptions) or recent asthma attacks. Participating practices were selected to represent inner city and rural practices and practices serving affluent and less affluent populations in line with convenience sampling. The children and their carer(s) were invited for an asthma review by their practice by text message or by telephone call and offered the opportunity to take part in the study. After obtaining written informed consent, participating children were issued with a Hailie® smart inhaler by the Study Nurse who conducted a 3-month phone call to check asthma control and feed-back on the adherence data based on information available on the Hailie online platform. All families and CYPs were invited for a face-to-face review including lung function testing at 6 months. The total time that a child remained in the study was 6 months from the issue date of the digital smart inhaler.

All the parents of children who had participated in the main study were subsequently approached by the study research nurse or technical support team (either in person, through email or telephone) to confirm willingness to take part in the qualitative study. Interested participants were given the Participant Information Sheet (PIS) which set out the researcher objectives and interest for the study, and linked up with the qualitative researchers, IQ and MG, who then obtained informed consent. Children (>11 years) and parents were offered the option take part in individual or joint interviews. Healthcare providers from the participating primary care surgeries were invited by the trial team and interested HCPs were linked to the qualitative researchers who followed a similar procedure to formally recruit them into the study.

We had planned to recruit approximately 30 participants (10% of the proposed sample in the main study) which we believed would enable us to achieve data saturation and also give our sample sufficient ‘information power’ to answer the research questions, as suggested in the literature.^9,10,11^

Interviews were conducted between July and December 2024 with parents, children and HCPs either online using Microsoft Teams or face-to-face depending on participant preference. Interviews were conducted in English by either IQ or MG (who both hold relevant PhDs and interests in health services as professional researchers) using separate topic guides for parents, children and healthcare professionals. Topic guides were developed in consultation with members of an existing paediatric asthma Public and Patient Involvement and Engagement (PPIE) group and key topics covered relate to experiences of paediatric asthma care and management broadly and digital technology and the experience specifically with the use of the Hailie® smart inhaler. Discussions took approximately 45 min. Following their participation, a £20 gift voucher was given to each participant in recognition of their contribution to the research. Interviews were recorded with permission and transcribed verbatim. Anonymised transcripts were checked for accuracy by the research team prior to analysis. The data were subsequently thematically analysed by IQ and MG.^12^ We aimed for and achieved thematic saturation at the analysis stage wherein no new codes and themes emerged from the collected data.^9^

## Results

Of the total of 72 children who participated in the smart inhaler study six parent–child dyads and one parent took part in the interviews. Demographic details of patient participants are provided in Table 1. Additionally, three HCPs (1 Nurse, 1 Pharmacist and 1 Practice Manager) involved in paediatric asthma care and/or management were also interviewed (see Table 2). A ‘practice manager’ is usually a non-clinical member of the staff and is responsible for operational and business matters in primary care settings. We included non-clinical staff, who had been involved in setting up the study, into the nested qualitative study to understand what service (re)arrangements are needed to implement digital smart inhaler monitoring in primary care.

**Table 1.** Parent and child participants’ demographic data.

Parent ID	Age range	Gender (relation to child)	Ethnicity	Languages spoken at home	Employment	CYP ID	CYP age
P1	40–49 yrs	F (mother)	White British	English	Part-time employed	CYP1	8Y
P2	40–49 yrs	M (father)	White British	English	Full-time employed	CYP2	12Y
P3	-	F (mother)	-	-	-	CYP3	8Y
P4	40–49 yrs	F (mother)	Black African	English, Portuguese Swahili	Part-time employed	-	7Y
P5	20–29 yrs	F(mother)	White British	English	Full-time employed	CYP5	10Y
P6	>50 yrs	F (mother)	White British	English	Part-time employed	CYP6	13Y
P7	40–49 yrs	F (mother)	Asian	English Tamil	Not in employment	CYP7	7Y

**Table 2.** Healthcare worker demographic data.

Participant ID	Age range	Gender	Role	Years working in paediatric asthma
HCW 1	20–29 yrs	Female	Pharmacist	1.5 years
HCW2	30–39 yrs	Male	Practice manager	18 years
HCW3	>50 yrs	Female	Children's respiratory specialist nurse	28 years

Two specific themes regarding the use of the Hailie® smart inhaler technology were identified. The first theme is the positive responses to the smart inhaler and its related technology and the second theme relates to technical aspects and areas for improvement.

## Positive responses to the inhaler and its related technology

This theme demonstrated participants’ positive responses in their experience of using the Hailie® smart inhaler and its potential acceptability. Several relevant subthemes were identified, as follows:

### Accepting the concept

Participants appreciated the idea of using digital technology and a Hailie® smart inhaler and were supportive of its development:

I think it is a really good idea. It's really helpful and useful to be able to track it and make sure, you know, I go to work quite early, to be able to look at my app and know she's had it [the dose of medication] in the morning, that would be really good peace of mind for me just to know she's had it. (P5)

So I think when it comes to children, most of the kids are much more smarter than I was when I was five or seven years old. I had no idea what was going on. And these kids nowadays they have iPhones and iPads and everything so they are much more savvy, they know what's going on. (HCW2)

Yeah, all of them, because I could see how frequent I was using it, I could see how much I was using it and that actually it made me realise a few things that I didn’t know, that I didn’t understand before about how inhalers work. (P4)

Obviously it's useful for them because I think they get reminders, the app, so they can track it. So it's a bit better for parents to work with their kids to see if they’re adhering. (HCW1)

### Utility

Participants also commented on the usefulness of the smart inhaler and described how easy it was to use and its effectiveness in enabling them to monitor their children's inhaler usage:

Obviously the new inhaler device is good because it will monitor it [their asthma] for them. Inhalers that have the counters on, particularly the newer inhalers where it counts down every one puff is useful. Because if they’re like “Oh I’m not sure” it's visually going to tell them “your count has gone down”. (HCW1)

Yeah, no, they’ve been good in the fact that it enables me to see that she's been taking them, because sometimes I trust her and I say ‘right, I'm going to go downstairs and, I don’t know, get lunch or whatever, you take your inhaler’ and then I could check on the app that she was taking them. (P1)

I think it's just quite interesting to see my patterns of taking it and not taking it and maybe certain times in say a month that you can see whether I've had to take my blue inhaler more and whether that's because I've been to someone's house or there's more triggers, or whether it's just that I've maybe dropped off a bit on my brown inhaler. (CYP2)

For me as a parent it's a really good way of making sure that you are on top of it because although we keep the inhalers by the sink so in the morning he brushes his teeth, the inhalers are there…. So if there's any sort of thing where I say ‘have you brushed your teeth and done your inhaler’ and if he says ‘yes’ I can check on the app. And that is quite a useful tool!. (P3)

## Areas for improvement

This theme captured the experience with the technology encountered by the participants’ whilst using the Hailie® smart inhaler as well as their suggestions for improving the overall experience for future users. Within this theme, two subthemes were detailed, as follows:

### Synching challenges

Some parents reported that the devices were not always synchronising accurately with the amount of dosage that had actually been taken by the child in relation to what was showing on the app:

They (the Hailie® smart inhaler) weren't always synching up properly, so sometimes I would watch her do two, but it would only read as one on the app and I'm like oh OK, sometimes it would take a while to synch. (P4)

It wouldn’t always register that he’d taken it and there was a few days went past and it was saying he wasn’t having it and then I got some calls from the company just saying ‘it looks like [Name 2]'s not taking his inhaler’ and I kept saying ‘I can assure you he is, it's not registering’. (P3)

They didn’t work too well for me unfortunately. They didn’t sync properly. We did have to manually sync them a lot it didn’t seem to go through. It didn’t record unfortunately really on the app either when she’d used the inhalers, it never really showed. (P5)

If the person on whose phone the app was installed was not home at the time when the doses are taken, this would not register. Remote syncing was a challenge. (P7)

### Suggestions for improvement

Participants suggested ways in which the device and its related technology may be improved for future users:

Perhaps if there was something where you could earn rewards on an app or you get a character that you could customise or something like that. I think there's ways that you could definitely make it more appealing to children. (P3)

If it gave you any indication of how long you should be holding your breath for or when to take a puff. (P2)

I guess perhaps from an app point of view, yeah, identifying if she hadn’t taken any doses in a particular day, if you got a notification to say, you know, maybe just check that. (P2)

Digital inhaler only beeped once during the day, would have been helpful to get two reminders. (P6)

One HCP mentioned that while the technology is beneficial, its effectiveness depends on close monitoring and support from the clinical team.

I’ve been involved in digital inhaler monitoring for two or three years now, before this study. And I do see adherence improve for the first few weeks. But I think you need to keep on top of it. You need to support them [families] by not just, it's almost like we’re putting these devices on these inhalers to monitor them but also to encourage adherence. So I think you can’t just send them away with it and expect them to do it on a day to day basis for weeks on end. You need to support them with calls, with support, with assurance, things like that. And also it's updating asthma action plans, encouraging them to re-address it in clinics and things like that. (HCW3)

## Discussion

Findings from this qualitative study highlight the benefits of using digital inhaler technology from the perspective of carers looking after children and young people with asthma and from the CYP themselves. A considerable amount of literature on technology supported asthma management exists^3^ but the views of carers and CYP are rarely represented in the asthma literature. The digital inhaler technology in the management of asthma includes helping carers/CYP to self-manage the inhalation quality and adherence by providing them with feedback on inhalation parameters and controller medication usage in real time on their smartphone App (13). This is the first study considering the use of the Hailie® smart inhaler in CYP and parents/carers identified in primary care were the majority of patients with asthma are managed.^9^ Participants were from a diverse range of socioeconomic backgrounds. Parents and carers in our study reported high user acceptability and increased adherence to medication following the use of the Hailie® smart inhaler. It is likely that the growing use of telemedicine after the pandemic has led to increased willingness of parents/carers and CYP to use digital tools for asthma management.^13^ Most participants in this study did see the positive potential in this area.

Digital monitoring can be empowering for families and CYPs to manage their/their child's condition and improve adherence.^3^ Furthermore, preliminary randomised studies also suggest that patients using such devices have greater odds of clinically meaningful asthma control improvements.^14^ However, as suggested by one of our HCP participants and also emphasised in other studies, offering patient support and education is critical for the effectiveness and sustainability of these digital interventions.^13,15^ Further research on the Hailie® smart inhaler usefulness in clinical management of paediatric asthma care in primary care should be conducted. Our findings also call for greater collaboration between HCPs and industry professionals to develop a wider offer of support, education and efficient technologies for asthma patient utilisation.

Digital smart inhalers are currently not in routine use in primary or secondary asthma care in the UK but they are occasionally available in severe asthma services. The reason for this is that there is no National Health Service (NHS) reimbursement for these devices because their cost-effectiveness has not been conclusively demonstrated. There are also technical issues that require resolving, requiring more robust research in this area. Nevertheless, clinical improvements have frequently been shown in studies using digital smart inhaler monitoring and it is likely that as part of the digital transformation in the NHS this type of technology will become part of routine care. Integration of digital smart inhaler data into General Practice (GP) computer systems for seamless access to the data could also boost HCP enthusiasm for this technology.

Our study has limitations; firstly, as it was a self-selected sample, there is a likelihood of response bias. However, we have tried to limit this bias during the interviews by probing both positive and negative views and experiences of our participants. Secondly, our HCP sample was small which made it challenging to analyse fully the service aspects of implementing digital asthma monitoring in primary care and will need further exploration to understand the providers’ perception.

## Conclusion

Our study demonstrates that there is acceptance of digital asthma monitoring among patients, their families and HCPs involved in paediatric asthma care in primary care in the UK. Digital monitoring has been particularly appreciated by patients and families for its utility in providing medication reminders which has helped with adherence. Successful integration relies on addressing challenges such as embedding the technology into clinical workflows, ensuring adequate education for both clinicians and patients, and providing accessible user support. Empathetic communication and guidance from the clinical team can also help build confidence and encourage uptake of digital asthma monitoring. Embedding a continuous improvement process – where technology evolves in response to user feedback, clinical needs, and real-world use – can further support sustained adoption and effective integration into routine care.

## Appendix A

Digital smart inhaler asthma management in children and young people

### Parent/carer's demographic details survey and interview topic guide

Participant ID Number: ________________________ (To be filled by research team)

*Prior to the interview and the questionnaire/survey, participants will be re-reminded (as also detailed within the participants information sheet and participants consent form) that they do not have to answer any question if they do not want to and are able to finish at any point.*

1. Age
  <20 yrs □ 20–29 yrs □ 30–39 yrs □ 40–49 yrs □
  >50 yrs □
2. Gender (please tick) Male □ Female □ Other □
3. Relationship with the child_________________________________
4. How would you rate your child's asthma control status?
  Very well controlled □ Well controlled □
  Not very well-controlled □ Not at all well controlled □
5. Ethnicity (please tick)
  White Black or Black British
  British □ Caribbean □
  Irish □ African □
  Any other White background □ Any other Black background □
  (Please specify)__________________ (Please specify)_____________
  Mixed Asian or British Asian
  White and Black Caribbean □ Indian □
  White and Black African □ Pakistani □
  White and Asian □ Bangladeshi □
  Any other mixed background □ Chinese □
  (Please specify)__________________ Any other Asian background □
  (Please specify)_____________
  • ther Ethnic groups
  Arab □ Prefer not to say □
  Any other ethnic group □
  (Please specify)__________________
6. Were you born in the UK?
  Yes □ No □ Prefer not to say □
7. Language(s) spoken at home
  (a)____________________ (b)____________________ (c)___________________
8. Home postcode (please only specify the first part)________________________
9. Employment status
  (a) Full-time employed □ (b) Part-time employed □
  (c) Self-employed □ (d) Not employed □
  (e) Student □ (f) Retired □
10. Number of household members___________________________
11. Number of children below the age of 16 years _________________
12. Number of children in the household with asthma____________________

Qualitative open-ended interview questions:

1. **Introductions**
  (a) Names, who we are, where we’re from
  (b) Seek consent to continue and to record the interview (if applicable).
  (c) Let them know that no personal identifiable data will be recorded and a participant number will be allocated to them
2. **Family background**
  (a) Please tell me about yourself and your family. Does anyone else in your family have asthma?
  (b) Can you tell us about your child's health and daily routine? (Going to school; levels of physical activity; any other ailments/conditions)
  (c) In the demographic form you rated your child's asthma control status as____. Could you explain why?
3. **Exploring understanding and beliefs about asthma and its impact**
  (a) When did your child's asthma start? Can you describe the signs and symptoms that you noticed before your child was diagnosed with asthma? What did you know of asthma before your child was diagnosed?
  (b) Can you please share your experiences of getting an asthma diagnosis for your child?
  (c) What do you think are the factors that caused your child's asthma? What do you think triggers their asthma? (Air pollution? Temperatures? Environments? Exercises? Activities? Stress? Food/drink?)
  (d) In what ways has asthma affected your child's life? (Physical activity? School attendance? Social life?)
  (e) In what ways has asthma affected you/your family's life? (Social life? Balancing care and work? Making structural/building changes in the house)
4. **Exploring asthma care and management**
  (a) Can you please tell us what advice have you received from the doctor regarding managing your child's asthma (including medicine adherence)? How has this helped you in understanding and managing your child's asthma? (Probe: Did you feel you needed more support or information?)
  (b) How often do you see your child's doctor/nurse about their asthma (annual reviews)? How helpful do you think are these appointments for you in understanding and managing your child's asthma?
  (c) Can you tell us about your experience of getting clinical help for your child's condition/breathing? (including accessibility, perceived relevance, patient-centredness)
  (d) How often are you recommended lung function tests for your child? What has been your experience of these tests? Do you feel the tests help you to understand or manage your child's asthma?
  (e) Has there been any time(s) when you had to seek urgent care for your child's asthma? If yes, could you please share the episode(s)
  (f) Could you describe how you or your child manage their asthma on a day-to-day basis (medications, inhalers, restrictions on physical activity, complementary therapies, home remedies)?
  (g) Have you observed anything which makes your child's asthma worse? What do you do in circumstances when your child's asthma worsens?
  (h) Have you observed anything which makes their condition/breathing better?
5. **Understanding their perspectives on digital asthma smart inhalers**
  (a) Can you tell me about your experience of using the digital inhaler? (being fitted, instructions received)
  (b) Why did you choose to try the digital inhaler?
  (c) From your usage, are there any features of the digital inhaler that you have found helpful?
  (d) Are there any features of the digital inhaler that you have found challenging to use?
  (e) If you could change or add anything about how the technology works, what would that be?
6. **Recommendation for improved practice**
  (a) What do you believe could help you better manage or improve your child's asthma condition (Housing? Behaviours? God? The Doctor? Anything else)
  (b) Is there anything you would like to change about the help/healthcare that your child is receiving?
  (c) What would an ideal asthma service look like for you/your child’ (would you prefer more regular asthma reviews, would you like access to an advice line?)?

**Anything not covered?** Is there anything that we haven’t covered in the interview that you think we should know or think about?

**Closing and thanks** – check that the participant is still happy for you to use all the information provided. Thank them for their time and contribution and remind them about the voucher.

## Appendix B

Digital Smart Inhaler Asthma Management in Children and Young People

### Children and young people's interview topic guide

Participant ID Number: ________________________ (To be filled by research team)

*Prior to the interview and the questionnaire/survey, participants will be re-reminded (as also detailed within the participants information sheet and participants consent form) that they do not have to answer any question if they do not want to and are able to finish at any point.*

[Demographic Information to be sourced from the SBRI Data Capture Sheet]

Qualitative open-ended interview questions:

1. **Introductions:**
  (a) Names, who we are, where we’re from
  (b) Seek consent to continue and to record the interview (if applicable).
  (c) Let them know that no personal identifiable data will be recorded and a participant number will be allocated to them
2. **Icebreakers**
  (a) Favourite colours/games/one interesting fact about yourself/passion that you love to do
3. **Exploring understanding and beliefs about asthma and its impact**
  (a) Can you tell us about your breathing problems? (how long, how frequent, symptoms) Do you know that you have asthma? Do you know what asthma is? Did you know of asthma before you were diagnosed?
  (b) What do you think have caused your asthma? What do you think triggers your asthma? (Air pollution? Temperatures? Environments? Exercises? Activities? Stress? Food/drink?)
  (c) Does your asthma prevent you from doing certain things? (Physical activity? School attendance? Social life?)
  (d) How does your asthma make you feel (both physically and mentally)? Is there anything that particularly worries you?
4. **Exploring asthma care and management**
  (a) Can you tell me what the doctor has told you about your asthma and how to manage it? How has this helped you in understanding and managing your asthma? (Probe: Did you feel you needed more support or information?)
  (b) How often do you see your doctor/nurse about your asthma? (annual reviews) How helpful do you think are these appointments for you in understanding and managing your asthma?
  (c) Can you tell me about your experience of getting medical help for your condition/breathing? (how easy it was for you to get help, did you feel supported, did you feel cared for)
  (d) Have you undergone any lung function tests (spirometry, bronchodilator reversibility and FeNO)? If yes, what has been your experience of these tests? How do you feel about doing these tests? Do you feel the tests help you to understand or manage your asthma?
  (e) Has there been any time(s) when you had a severe asthma attack? If yes, could you please share about the episode(s)?
  (f) Can you describe how you manage your asthma on a day-to-day basis? (medications, inhalers, restrictions on physical activity)
  (g) Can you tell us who helps you in managing your asthma in your home?
  (h) Have you observed anything which makes your asthma worse? What do you do in situations when your asthma worsens?
  (i) Have you observed anything which makes your condition/breathing better?
5. **Understanding their perspectives on digital asthma smart inhalers**
  (a) Can you tell me about your experience of using the digital inhaler? (being fitted, instructions received)
  (b) From your use, are there any things/features of the digital inhaler that you liked better?
  (c) Are there any features of the digital inhaler that you have found tricky to use?
  (d) If you could change or add anything about how the digital inhaler works, what would that be?
6. **Recommendation for improved practice**
  (a) What do you believe could help you better manage or improve your asthma condition?’ (Housing? behaviours? God? the doctor? anything else)
  (b) Is there anything you would like to change about the help/healthcare that you are receiving?
  (c) What would an ideal asthma service look like for you (e.g., would you prefer more regular asthma reviews, would you like access to an advice line)?

**Anything not covered?** Is there anything that we haven’t covered in the interview that you think we should know or think about?

**Closing and thanks** – check that the participant is still happy for you to use all the information provided. Thank them for their time and contribution and remind them about the voucher.

## Appendix C

Digital Smart Inhaler Asthma Management in Children and Young People

### Healthcare worker demographic data form and interview topic guide

Participant ID Number: ________________________ (To be filled by research team)

*Prior to the interview and the questionnaire/survey, participants will be re-reminded (as also detailed within the participants information sheet and participants consent form) that they do not have to answer any question if they do not want to and are able to finish at any point.*

13. Age
  <20 yrs □ 20–29 yrs □ 30–39 yrs □ 40–49 yrs □
  >50 yrs □
14. Gender (please tick) Male □ Female □ Other □
15. Healthcare Role________________________
16. Years working with children with asthma________________________________
17. How many paediatric asthma cases do you see in a month?___________________

Qualitative open-ended interview questions:

**Introductions:**

(a) Names, who we are, where we’re from
(b) Seek consent to continue and to record the interview (if applicable).
(c) Let them know that no personal identifiable data will be recorded and a participant number will be allocated to them

1. BACKGROUND
  (a) To start could you introduce yourself and your clinical role?
  (b) How long have you worked in paediatric asthma care?
  (c) How often do you see children with asthma?
2. CLINICAL GUIDANCE AND PRACTISE
  (a) Could you please share what clinical guidelines do you follow for offering treatment/care to paediatric asthma patients? Could you kindly elaborate how do these guidelines help or challenge you while carrying out your practise?
  (b) Do you use personalised written asthma action plans in your practise? Can you please share your experience of using these plans?
  (c) How often do you use objective lung function tests in your practise? What is your opinion on the usefulness of these tests?
  (d) How often do you come across salbutamol overuse among patients? How do you attend to such cases?
  (e) How often do you check for adherence to preventer medication? How do you feel about checking for adherence? How do you attend to cases where you become aware of non-adherence?
  (f) Do you currently make use of any decision-making support tool or electronic technologies in diagnosis or treatment of asthma in children? If yes, could you kindly share you experience of those?
3. FAMILY CONSIDERATIONS
  (a) What do you see as the main aims of the treatment/management you provide?
  (b) What advice or information do you offer to families with children suffering from asthma?
  (c) What are the common concerns or questions that families have regarding management of their child's asthma?
  (d) What are some of the main concerns of the children?
4. CHALLENGES AND RECOMMENDATIONS
  (a) What would you describe as some of the most challenging aspect of treating/managing paediatric asthma? (Communication challenges, non-attendance of asthma reviews, limited staff)
  (b) What factors do you think support or hinder parents in managing their child's asthma?
  (c) What factors do you think support or hinder children in managing their own asthma?
  (d) What would be your recommendations/solutions for addressing the challenges or barriers that you mentioned?

**Anything not covered?** Is there anything that we haven’t covered in the interview that you think we should know or think about?

**Closing and thanks** – check that the participant is still happy for you to use all the information provided. Thank them for their time and contribution and remind them about the voucher.
